# Supplementary material for: The effect of bio-banding on academy soccer player passing networks: Implications of relative pitch size
Source: PLoS One. 2021 Dec 16;16(12):e0260867. doi: 10.1371/journal.pone.0260867 (PMC8675666; doi:10.1371/journal.pone.0260867)
Supplement: Attachment — Submitted filename: Comments to Editor and Authors .docx [file pone.0260867.s001.docx]

Dear Editor in-Chief,

I would like to thanks for the opportunity to revise this article entitled “The effect of bio-banding on academy soccer player passing networks: implications of relative pitch size”.

In academy players, the use of bio-bending is a very interesting aspect to deliver a very individualized training prescription. However, it is not easy to find the right way to apply all this information into practice due to a mandatory chronological classification for championship participation (U19, U18, U17, etc). As such, future perspectives are required and I would like to thanks the authors for their contribution.

The article is well written and easy to understand. However, honestly, I find the practical applications quite weak. Firstly, the results about the effects of pitch size on technical aspects (increase of technical actions) are well acknowledged. Secondly, the use of bio-bending analysis integrated with technical-tactical analysis require a lot of work to be well determined within training routine. Therefore, I would to highlighted the great work performed by the Authors but I would to invite them to reworded and rethink the use of these information as ‘game-changer information’. Could these information and analysis change the approach to selection and increase the quality of training? Could the use of bio-bending analysis integrated to SSGs provide the opportunity to increase the training quality and performance development of the youth players? In my opinion the work required to create these specific analyses inside elite academy training routine should be more highlighted. I would kindly ask to the Authors to revise their paper (discussion, conclusion and practical applications) considering the aforementioned questions, please. As practitioners, reading this paper I need to be convinced that is mandatory required this analysis and that I need to involve practitioners daily to analyze this information in practice to increase the quality of performance development in elite academies (I suggest to mainly consider passing metric network than pitch-size).

Introduction:

Line 70: please provide some previous findings about how the readers could apply bio-banding information to increase the quality of their work.

Line 76 to 89: this paragraph is not clear. Please, try to simplify

Line 86-89: If the authors would to introduce the effects of SSG pitch size I believe that could be interesting to briefly speak about effects of pitch size on tactical, physical and physiological aspect as previously stated for example in Olthof et al JSS 2018, Riboli et al plos one 2020, Castagna et al IJSPP 2019, etc..

Materials and methods

Line 148: In my opinion would be useful to provide here how long each SSG lasted (as reported at line 157)

Line 190: in my opinion could be interesting to provide a representative figure (graphical representation) of network analysis to support figure and equations (are these latter mandatories required?)

Discussion:

I believe that the discussion and especially conclusion and practical applications should be more based on the application of these results and it should convince the readers about the effective needs to increase the integration of bio-banding analysis with network analysis into daily practice, as previously suggested. Please, provide why this information could be ‘game-changer’ information and affect training prescriptions on daily base. The Authors could convince readers that is very important to integrate all these analysis into daily practice, not only suggest to play with three late-maturing and one early-maturing player. This paper could have great new practical applications opening to great future perspective for training prescriptions but the author should suggest these solutions, please.
