# Supplementary material for: The effect of bio-banding on academy soccer player passing networks: Implications of relative pitch size
Source: PLoS One. 2021 Dec 16;16(12):e0260867. doi: 10.1371/journal.pone.0260867 (PMC8675666; doi:10.1371/journal.pone.0260867)
Supplement: Attachment — Submitted filename: Revision 1.docx [file pone.0260867.s002.docx]

I would like to thank the editors for the opportunity of reviewing this manuscript. This work aims to examine the effect of bio-banding players on passing networks creating during 4-a-side games. I find this work very interesting and relevant. I think it is, in general, well written. However, I think there are several weaknesses (mainly in methods) that need to be addressed before considering this work for publication. I hope my comments help to improve the quality of this manuscript. I am also happy to discuss my comments with the authors in case they disagree.

**ABSTRACT**

I suggest the authors go straight to the study’s objective, which in fact are three, not two, as stated in the abstract.

**Methods:** I think they are not clear and need to be improved. It is not clear anything related to pitch size nor a subjective-coach-based scoring system.

**Results:** I think they are described chaotically and are not easy to understand by the reader

**Conclusion:** I suggest the authors go straight to the point, so they might save some words for describing the methods more accurately.

**KEYWORD:** avoid using the same words as in the title. For instance, soccer might be replaced by football.

**INTRODUCCTION**

**Comment 1:** I like the way the introduction of the paper has been written. However, I think it might be appropriate to provide further comments on the need of evaluating pitch size. Moreover, the statement in lines 116-117 requires a reference.

**Comment 2:** I also think it might be appropriate to provide further information to justify why the comparison between network analysis and subjective-coach-based scoring system is required.

**METHODS**

***Participants***

I would like to know why maturation status was only measured using height. Why was not participants’ sexual development evaluated by other instruments like the Tanner Scale?

***Experimental design***

I do not think this is clear. I read this section several times and I have still doubts. I encourage the authors to rewrite this section to facilitate understanding.

**Lines 145-146:** According to this statement, only 2 Early teams and 2 Late teams were created for this study (in total 16 players participated). This is not clear, as 44 players were recruited in total (24 from team 1, and 20 from team 2). Also, it is not clear if players from both teams were mixed or not. it is not clear either who created each 4-a-side team. Were they created randomly only considering the bio-banding score? Were participants gathered into their team according to coach criteria? Finally, if only 16 players per team participated what happened with the remaining players? If all players participated, can this fact bias the outcomes?

**Lines 146-147:** I know the term “round robin”, and to my knowledge, it just means all teams play each other once. I might be wrong as I am not English, but I encourage the authors to be specific and clearly state that each team played against the other three teams a total of two times. Otherwise, I cannot understand how each team played 6 games.

**Lines 147-148:** I understand the three game’s types but, I think it should be appropriate to state that Early vs Early and Late vs Late were played two times. On the contrary, Early vs Late was played four times.

**146-148:** Which were the matches order? were they always the same? I think it might bias the outcomes.

**Line 148-149:** Why were 3 minutes of passive recovery set? I am not sure it is enough to limit fatigue. Is there a reference that can be used to justify this? Please, notice that if 6 matches were played in total players played for 30 minutes. Fatigue might exist in last two or three games.

**Lines 149-150:** It is not clear at all the players re-assignation. I think it might be easier to understand if the authors explain at the beginning of this section that two different tests were conducted. Firstly, players were gathered into a team according to their bio-banding score, while secondly games were conducted gathering two early players with two late players in each team.

**Lines 150-152:** Why were 6 matches played in “mixed”? This fact increases the number of observations of mixed games while the other three games observations are much lower. Also, I guess chronological do not always meet the criteria of 2 Early players and 2 Late players. So, might this fact bias the outcomes?

**152-154:** How far was each goal from each other and from touch line?

**Line 158-162:** I think it should be appropriate to inform at the beginning of this section that in total four different 4-a-side games were played. There is a lot of information here. Maybe it might be useful to use a Table or Figure to display part of this information in a clearer way.

**Lines 158-162:** Why were these pitch size selected? Why were they in square format instead of rectangle, which better represent a soccer field and the reference used to justify the dimension (20) used a rectangular field (18.3 m x 23.0 m).

**Line 158:** Why was only one test-day completed per week?

**Additional comments:** I think that further information is needed here. Was there some kind of control to avoid participants to be overload or fatigued before each test-day? Was diet controlled? When was the test-day conducted? Did participants tested for 24, 48 or 72-h before each test-day and did not engage in vigorous physical activity within this period? I think this is important as might bias the outcomes.

***Data collection***

**Lines 169-179:** I know PlayerMaker devices, but I am not too familiarised with them. According to the authors, this device can identify tactical information related to passing. I think I know how it works, but there are some important information missing:

1. how does the system distinguish between a failure pass or a shot to goal?
2. How accurate is PlayerMaker in collecting passing-related information?
3. Is this system validated for this study’s purpose?
4. Has been this system used in previous studies to analyse passing behaviour of soccer players?
5. Is this system also able to identify the distance of each player from each other?

**Line 180-186:** I also think there is quite a lot of information missing here:

1. How many coaches used the Game Technical Scoring Chart?
2. were they familiarised with this instrument?
3. **Line 186:** were some coaches only assessing one player?
4. Also, I wonder why this tool was selected given the fact it measures other behaviours besides passing.
5. Many readers will not be familiarised with the F.A. level so I think it should be clarified what means to be F.A. Level 1 coach.

**Results:**

**Line 247:** This first sentence is repeated. So, it should be removed from here or from methods.

**Line 249:** What does the number in brackets mean? Standard deviation? This fact is described later in the line so it should be mentioned earlier. Also, I think it might be appropriate to insert the symbol ±

**Line 251:** I think it might be good to remember that “game type” mean the (Early/Early, Early/Late, Late/Late and mixed).

**Lines 260-273 and Table 2:** in order to be consistent with the objectives and to facilitate the reading, I recommend the authors to first talk about the data from the network passing analysis and then data from Game Technical Scoring Chart. I think this is the order that the reader expects finding the information.

**Pooled:** Why “pooled” is used to mean mixed team instead of “mixed” as described in methods? This comment is also for the text.

**DISCUSSION**

**Lines 277-279:** I am not that sure about this statement. Bio-banding effect was only found in early players when moving from Early team to mixed-team. So, I think this first statement is not totally right. Actually, there is not comparison between early players and late players, comparisons described in results the comparison described are among game type and pitch size. Table two provide comparison between early and late players, but I am not sure what is being compared as this information is not explain in statistical analysis section. In any case, I do not see a direct comparison between early players and late players for the different studied variables.

**Line 279 (finding 1):** What is better and more effective collective behaviour? Higher number of passing? higher passing accurate? I do not think “better” is the right word, I would recommend use a different word. On the other hand, earlier players only showed a reduction in degree centrality, closeness centrality and increases in betweenness centrality when comparing their performance in bio-banding matches and mixed matches. The authors do not identify differences between early and late players in results. Accordingly, I do not think this statement is too accurate.

**Line 283:** I do not think pass attempts is a technical action, but a tactical behaviour. Network density and network intensity are not technical action either so, I do not think this statement is right.

**Lines 284-298:** I do not think this present study can be compared with the work of Cumming and Brown (18) or Bradley and Johnson (17) as these studies analysed players’ perception while the present work is analysing passing behaviour.

**Line 301-310:** I agree, but passing behaviour is not a technical action but a tactical response to the game demands. Passing is a tactical action not technical. Technical is kicking the ball, controlling the ball, etc.

**Additional comment:** I think the authors must disclose the main limitation of their study.

**CONCLUSIONS**

**Paragraph 1:** The authors should go straight to the point and display the main conclusion of their study. Most of the information in this paragraph does not belong to conclusion

**Paragraph 2:** I recommend the authors to add an additional subheading for this information named “Practical application” given this information does not belong to conclusion.

**Lines 365—370:** I am not sure this can be postulated. The authors did not investigate the behaviour of players when a team is made by 3 late players and 1 early player. Therefore, this assumption is not supported in this work.
